# Biogenic amine production by the wine *Lactobacillus brevis* IOEB 9809 in systems that partially mimic the gastrointestinal tract stress

**DOI:** 10.1186/1471-2180-12-247

**Published:** 2012-10-31

**Authors:** Pasquale Russo, Pilar Fernández de Palencia, Andrea Romano, María Fernández, Patrick Lucas, Giuseppe Spano, Paloma López

**Affiliations:** 1 Department of Molecular Microbiology and Infection Biology, Centro de Investigaciones Biológicas, C.S.I.C., Ramiro de Maeztu 9, Madrid 28040, Spain; 2 Department of Agriculture, Food and Environment Sciences, University of Foggia, Via Napoli 25, Foggia, 71100, Italy; 3 UMR Œnologie, INRA, ISVV, Université de Bordeaux, 210 chemin de Leysotte, CS50008, Villenave d’Ornon, 33882, France; 4 Instituto de Productos Lácteos de Asturias, CSIC, Carretera de Infiesto s/n, Villaviciosa, Asturias, 33300, Spain

**Keywords:** Biogenic amines, Lactic acid bacteria, Putrescine, Tyramine, Food safety, Food toxicity

## Abstract

**Background:**

Ingestion of fermented foods containing high levels of biogenic amines (BA) can be deleterious to human health. Less obvious is the threat posed by BA producing organisms contained within the food which, in principle, could form BA after ingestion even if the food product itself does not initially contain high BA levels. In this work we have investigated the production of tyramine and putrescine by *Lactobacillus brevis* IOEB 9809, of wine origin, under simulated gastrointestinal tract (GIT) conditions.

**Results:**

An *in vitro* model that simulates the normal physiological conditions in the human digestive tract, as well as Caco-2 epithelial human cell lines, was used to challenge *L. brevis* IOEB 9809, which produced both tyramine and putrescine under all conditions tested. In the presence of BA precursors and under mild gastric stress, a correlation between enhancement of bacterial survival and a synchronous transcriptional activation of the tyramine and putrescine biosynthetic pathways was detected. High levels of both BA were observed after exposure of the bacterium to Caco-2 cells.

**Conclusions:**

*L. brevis* IOEB 9809 can produce tyramine and putrescine under simulated human digestive tract conditions. The results indicate that BA production may be a mechanism that increases bacterial survival under gastric stress.

## Background

Biogenic amines (BA) are natural toxins that can occur in fermented foods and beverages and may cause adverse health effects [1-3]. BA production in foodstuffs is mainly due to microbial metabolism of amino acids, with lactic acid bacteria (LAB) being the primary agents [4]. Tyramine and putrescine are the BA most frequently encountered [5].

*Lactobacillus* and *Enterococcus* spp. are often implicated in tyramine formation resulting from tyrosine decarboxylation [6-8]. Tyramine production has been observed in cheeses, fermented sausages and beverages [reviewed by 2, 3] and factors that influence tyramine biosynthesis have been reported [9,10]. A relationship between tyramine content of foods, and illnesses after ingestion, has been established [reviewed by 2]. These illnesses include headache, migraine, neurological disorders, nausea, vomiting, respiratory disorders and hypertension. Moreover, the adherence of some enteropathogens, such as *Escherichia coli* O157:H7, to intestinal mucosa is increased in the presence of tyramine [11]. Bacteria can produce putrescine from ornithine, using ornithine decarboxylase [12], or, alternatively from agmatine, using agmatine deiminase [13,14]. Putrescine synthesis was initially observed mainly in *Enterobacteriacea*, though recently it has been shown that LAB present in food and beverages can produce this BA [reviewed by 2]. Amines, such as putrescine, can react with nitrite to form nitrosamines, which can have carcinogenic properties and are therefore a potential health hazard to humans [3].

One open question is whether BA-producers present in fermented foods and beverages are able to survive in the human GIT and still produce BA. During digestion, the pH of the human gastric environment can decrease to values below pH 2. Some LAB possess high resistance to gastrointestinal stress and frequently have adhesive properties that allow them to colonize the intestinal tract [15]. We have recently shown that the dairy tyramine-producer *Enterococcus durans* 655 was significantly resistant to *in vitro* conditions which mimicked the human GIT and, it was able to synthesize BA under GIT stress conditions [16]. Possession of a functional tyramine biosynthetic pathway enhanced the binding of *E. durans* to Caco-2 human intestinal cells [16].

To further investigate this issue, we report here experiments with the wine strain *Lactobacillus brevis* IOEB 9809 [17], which possesses both the tyrosine decarboxylation and the agmatine deimination pathways [13,18,19]. Four genes (*tdc* operon) involved in tyrosine production have been identified in *L. brevis* IOEB 9809: a putative tyrosyl tRNA synthetase, a tyrosine decarboxylase, a tyrosine-tyramine exchanger and a Na+/H+ antiporter. The gene cluster for agmatine catabolism lies immediately downstream of the *tdc* operon, and its genes encode a putrescine transcarbamylase, an agmatine/putrescine exchanger, two putative agmatine deiminases (one of which, *aguA1*, encodes a catalytically active enzyme), a carbamate kinase and a putative transcriptional regulator (AguR). The presence of a functional substrate/product transmembrane exchanger in both systems suggests that the pathways may be involved in pH homeostasis.

In this study we have subjected *L. brevis* IOEB 9809 to an *in vitro* system, which partially mimics physical stresses in the human gastrointestinal tract, to determine if BA synthesis occurs. Transcriptional analysis was used to detect any enhancement of tyrosine decarboxylase (*tyrDC*) and agmatine deiminase (*aguA1*) gene expression. Furthermore, the adhesion of the IOEB 9809 strain to human epithelial intestinal cells was investigated and BA production in bacteria-human cells co-cultures was measured.

## Results and discussion

### Behaviour of *L. brevis* IOEB 9809 under simulated upper digestive tract conditions

To test for BA production and the influence of active BA biosynthetic pathways on bacterial survival IOEB 9809 was grown to approximately 8 × 10^8^ CFU mL^-1^ in MRS medium in the absence or presence of 10 mM tyrosine or 4.38 mM agmatine sulphate or both (these concentrations were previously found to be optimal for BA production; data not shown). Then, the cultures were subjected to conditions that simulated some of the more important conditions of the human upper digestive tract, including treatment with lysozyme at pH 6.5 (simulating saliva) and at a range of low pH in the presence of pepsin (simulating gastric stress). Acidity within the human stomach during digestion is in the range pH 1.3-3.5 which corresponds to the range of maximum activity of pepsin [20]. However, during food ingestion, and depending on the food matrix, bacteria can be exposed to a broader pH gradient. Therefore, during gastric treatment the bacteria were exposed to a decreasing range of pH from 5.0 to 1.8, which we have previously used for testing of probiotic and lactic acid bacteria [16,21-23].

**Figure 1:**
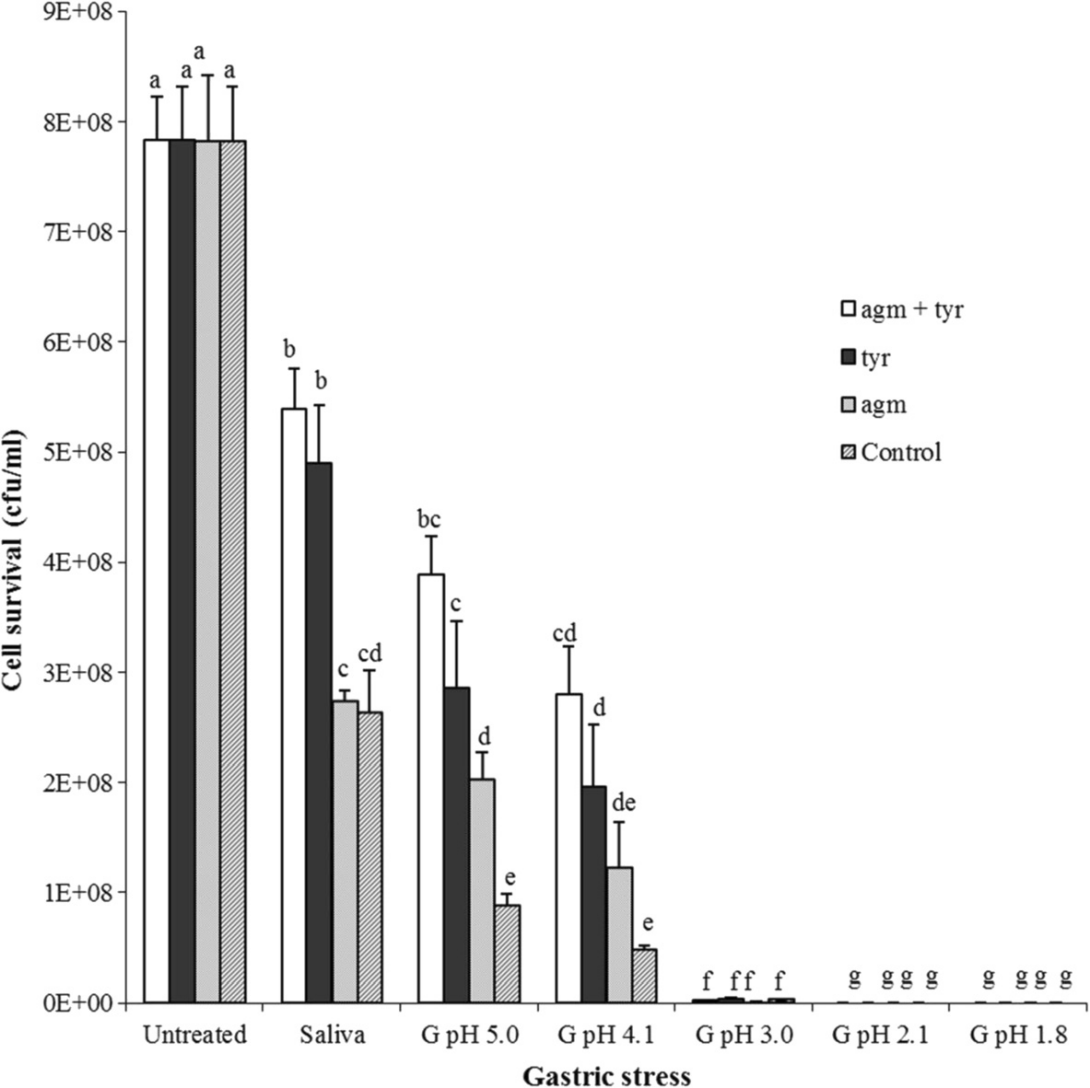
**Response of *L. brevis *IOEB 9809 to saliva and gastric stresses. **The salivary (saliva) and gastric (G) stresses were applied to bacteria in MRS (control), or in medium supplemented by addition of 4.38 mM agmatine (agm), 10 mM tyrosine (tyr), or both (agm + tyr). The values are the average of 3 independent experiments. Vertical bars represent the standard deviation. Differences were assessed by Anova test with all samples. Different superscript letters associated with values of CFU mL^-1^ indicate statistically significant differences (P < 0.05).

BA production was quantified by reverse-phase HPLC of culture supernatants, and cell viability was assessed by plate counting. Under all conditions, production of tyramine and putrescine was observed in the presence of the corresponding precursor (Table 1). The bacterium was sensitive to all conditions tested (Figure 1). The saliva simulation reduced the survival of IOEB 9809 to 34% in the control samples. A higher survival (62%) was observed in the presence of tyrosine, which was enhanced (69%) when agmatine was included in the assay. This survival-aiding influence of tyrosine was not previously observed with the dairy tyramine-producer *E. durans* 655 [16], and as far as we know this is the first report indicating that functional BA biosynthetic pathways or presence of their precursors contribute to diminish damage of cell wall by lysozyme. The mechanism for this is unclear.

**Table 1:** **Production of tyramine and putrescine by *L. brevis *IOEB 9809 in the presence of diverse BA precursors**

**BA precursor**	**Agmatine**	**Tyrosine**	**Agmatine +Tyrosine**	
**BA produced**	**Put (μM)**	**Tym (μM)**	**Put (μM)**	**Tym (μM)**
Saliva	22.33 ± 2.52^a^	26.08 ± 0.13^a^	32.66 ± 2.76^ab^	56.46 ± 3.06^ad^
G pH 5.0	37.67 ± 3.06^b^	78.29 ± 1.07^b^	57.27 ± 11.69^c^	194.63 ± 9.69^e^
G pH 4.1	36.00 ± 3.00^b^	122.30 ± 2.55^c^	39.22 ± 5.01^b^	174.46 ± 8.07^f^
G pH 3.0	11.59 ± 0.56^d^	82.18 ± 1.10^bc^	15.33 ± 1.05^da^	113.87 ± 5.27^c^
G pH 2.1	10.54 ± 0.46^d^	74.21 ± 1.07^bd^	14.32 ± 1.08^da^	76.10 ± 3.53^b^
G pH 1.8	11.21 ± 0.45^d^	62.26 ± 1.09^d^	13.42 ± 1.01^da^	50.91 ± 2.36^ad^

Tyramine (Tym) and putrescine (Put) production were detected by RP-HPLC during the saliva and gastric stress simulation in presence of 10 mM tyrosine, 4.38 mM agmatine or both. Results are expressed in μM of BA produced by 10^8^ CFU mL^-1^ in 20 min, they are the mean of three independent experiments and there are corrected for the CFU added to the experiment. Putrescine and tyramine were below the detection limits (2 nM and 2.5 nM) in the uninoculated MRS and in absence of the corresponding BA precursor. Differences were assessed by Anova test. Different superscript letters associated with values of the same BA indicate statistically significant differences (P < 0.05).

**Figure 2:**
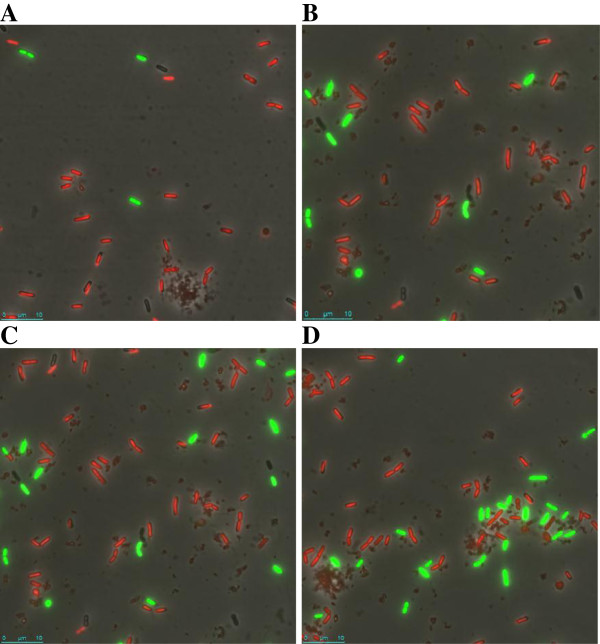
**Detection of live-dead bacteria by confocal microscopy. **Observation by confocal microscopy of *L. brevis *IOEB 9809 after gastric stress to pH 5.0 in absence of BA precursors **(A) **or in presence of: agmatine **(B)**, tyrosine **(C) **or agmatine plus tyrosine **(D)**. Green cells represent live bacteria, while red cells are bacteria with damaged membrane.

The pattern of increased survival was also detected under gastric simulation at pH 5.0 and 4.1. Below pH 4.1 reduction of viability was marked. This reduction was qualitatively confirmed by confocal microscopy, after bacterial staining with SYTO9 and propidium iodide. An example is depicted in Figure 2. In cultures subjected to gastric stress at pH 4.1 a mixed population of green (alive) and red (non-viable cells) were detected. Moreover, the proportion of green cells was low in the absence of precursors (Figure 2A) and progressively increased in the presence of agmatine (Figure 2B), tyrosine (Figure 2C) and both BA precursors (Figure 2D). In addition, in untreated cultures only green cells were detected whereas only a few cells, most of them red (non-viable) were observed after exposure to gastric conditions at very acidic pH 1.8 (results not shown). The tyrosine decarboxylase of IOEB 9809 has an optimal pH of 5.0 and is active between pH 3.0-7.0 in cell suspension [24]. In agreement we found the highest levels of tyramine production under gastric stress in the range pH 3.0-5.0 (Table 1). Interestingly, significant concentrations of tyramine (50 μM, 2.5 nmol mL^-1^ min^-1^) and putrescine (13 μM, 0.65 nmol mL^-1^ min^-1^) were observed in the samples exposed to pH 1.8 in the presence of the two BA precursors, even though only 1.7 × 10^1^ CFU mL^-1^ were detected at the end of the assay. This suggests that the inoculum was able to synthesise a substantial quantity of tyrosine decarboxylase during the test before cell death and lysis occurred, and that probably the tyrosine decarboxylase remained substantially active in the dead cells and cell lysate. The tyrosine decarboxylase of IOEB 9809 is active in a range of pH 2.0-8.0 in cell-free extract [24].

When we simulated the gastric environment, in addition to the action of lysozyme, the bacteria were subjected to multiple stress stimuli: decreasing pH, proteolytic activity of pepsin and heat shock at 37°C. Griswold et al. [25] (2006), propose that the *agdi* operon could be part of a general stress response pathway in *Streptococcus mutans*. The agmatine deimination, by forming ammonia and providing ATP, would result in mild deacidification of the medium, metabolic energy release and degradation of toxic compounds [25]. Here, the maximum levels of putrescine (around 40 μM) production by *L. brevis* were observed between pH 5.0-4.1 for cultures supplemented with agmatine (Table 1), which accords with that reported for *Lactobacillus hilgardii* at pH 4.5 [26] and for *Streptococcus mutans* at pH 4.0 [27].

There is evidence suggesting that BA production enables producing organisms to survive at low pH [28]. Our results show that at pH 5.0 the presence of agmatine, tyrosine or both precursors enhanced the cell survival two-, three- and four-fold respectively compared to controls (Figure 1). At pH 4.1, the beneficial effect on viability was even more pronounced (4- and 6-fold increase in the presence of tyrosine, and tyrosine plus agmatine); however, it has no beneficial effect at more acidic pHs (Figure 1). Thus, it seems that the beneficial effect of the putrescine and tyramine biosynthetic pathways is restricted only to mild acidic conditions.

### Transcriptional analysis of *tyrDC* and *aguA1* genes

**Figure 3:**
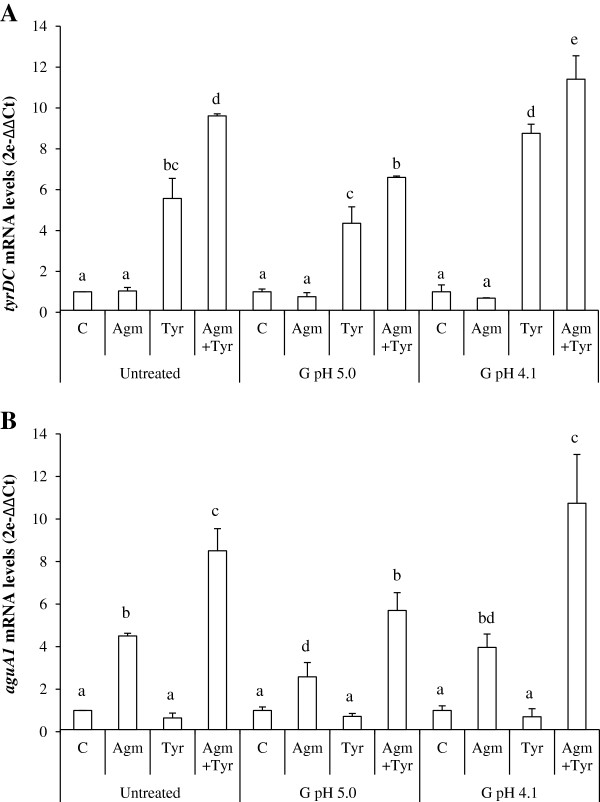
**Relative expression of *tdc *(A) and *aguA1 *(B) genes. **Total RNAs were extracted at mid-exponential phase prior treatment (untreated) and after saliva plus gastric stress at either pH 5.0 (G pH 5.0) or pH 4.1 (G pH 4.1), in presence of 4.38 mM agmatine, 10 mM tyrosine or both, or in their absence. mRNA levels were quantified as n-fold differences by comparing to RNA samples from their respective unstressed cultures (mRNA value=1). Relative levels of expression in absence of BA-precursors for untreated/G pH 5.0/G pH 4.1 were 1/0.7/0.4 in (A) and 1/0.6/0.3 in (B). Each experiment was performed in triplicate. Vertical bars represent the standard deviation. Differences were assessed by Anova test. Different superscript letters associated with values of either *tyrDC* or *aguA1* mRNA levels indicate statistically significant differences (P < 0.05).

The above results indicated that an increase of BA production occurred under saliva and mild gastric stresses, presumably due either to a physiological effect, or to increased gene expression. Therefore, expression of the *tyrDC* and *aguA1* genes, encoding tyrosine decarboxylase and agmatine deiminase, the key enzymes for tyrosine and putrescine synthesis [13,24], were analyzed in exponential phase cultures prior to, or after, exposure to saliva and gastric stresses at pH 5.0 or 4.1, in the presence or absence of BA precursors. Transcriptional levels were calculated relative to the mRNA levels of an unstressed sample for each condition tested, using the expression of the *tuf* gene as internal control (see Methods). A similar pattern of expression for both genes was observed in unstressed and stressed samples for all conditions tested (Figure 3). mRNAs corresponding to *tyrDC* (Figure 3A) or *aguA1* (Figure 3B) were induced only if the bacterium had been challenged with tyrosine or agmatine. Under all conditions tested, higher levels of *tyrDC* and *aguA1* transcripts were detected when both BAs precursors were present (approximately 9-fold increase in unstressed cultures and 11-fold under gastric stress at pH 4.1). Furthermore, it should be noted that transcriptional levels of the two genes in the control cultures were not reduced under conditions of gastric stress.

These results show a transcriptional induction of *tyrDC* and *aguA1* mediated by the respective BA-precursors under saliva and gastric stresses similar to that previously observed for IOEB 9809 under wine stress conditions [29]. The increased transcription of both genes in the presence of tyrosine plus agmatine strongly suggests a previously undetected synchronous regulation of both BA pathways, which deserves further investigation.

Considering the overall results pertaining to BA production (Table 1), cell survival (Figure 1) and transcriptional analysis (Figure 3), it appears that induction of BA biosynthetic pathway at the transcriptional level by the presence of the BA precursor under mild gastric conditions results in increase of the bacterial survival.

### Behaviour of *L. brevis* IOEB 9809 in the presence of human Caco-2 intestinal epithelial cells

**Figure 4:**
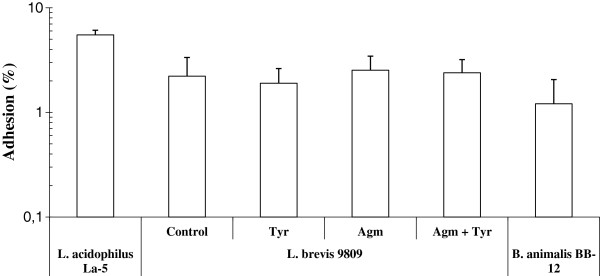
**Adhesion levels of *Lactobacillus brevis *IOEB 9809 to epithelial intestinal cells line. **Adhesion levels of *L. brevis *IOEB 9809, harvested at mid-exponential phase, to Caco-2 cells were measured after exposure in DMEM medium supplemented or not, with tyrosine, agmatine or both. Percentage of adhesion was normalized by using unwashed wells as control and compared with adhesion levels of probiotic strains *L. acidophilus* La-5 and *B. animalis *subsp. *lactis *BB-12. Each experiment was performed in triplicate. Vertical bars represent the standard deviation.

Our results revealed that at pH 4.1 there is an approximately 35% survival of IOEB 9809 (in the presence of agmatine and tyramine) and an approximately 0.4% survival at pH 3.0 (Figure 1). It is therefore possible, assuming ingestion of a high concentration IOEB 9809, that viable cells could be released from the stomach at the upper end of its normal pH range. Therefore, binding ability as well as production of BAs during co-incubation of IOEB 9809 with Caco-2 cells was analyzed. Caco-2 cells are human colonic adenocarcinoma cells that, after differentiation, have features characteristic of mature small intestine cells [30]. The maximum adhesion levels were obtained within the ratios of 1:100 to 1:1000 Caco-2 cells to bacteria after 1 h incubation, as we have also observed for other LAB and bifidobacteria [21,23]. Figure 4 depicts the results obtained with a ratio of 1:100, adhesion levels ranged from 2 to 3% approximately, values similar to the two probiotic bacteria tested *Lactobacillus acidophilus* La-5 and *Bifidobacterium animalis* subsp. *lactis* BB-12 (Figure 4). Moreover, we did not detect any statistically significant influence of the BA precursors on the adhesion capability of *L. brevis* (result not shown). Logically, the ability to adhere to the epithelium of the small intestine could be an aid to colonisation.

In addition, the bacteria could synthesize BA in the intestinal environment, and to test this hypothesis, the production of BA by IOEB 9809 in the presence of Caco-2 cells was investigated. The bacterium was exposed to the cells at a ratio of 1:1000 in DMEM medium for 8 h, in the presence or absence of the BA precursors, and the supernatants were analyzed by HPLC. Both BA were detected only when the precursors were present (Table 2 and data not shown). Levels of tyramine (180 μM) slightly increased in the presence of both BAs precursors (230 μM), and high levels of putrescine (1330–1980 μM) were observed irrespectively of tyrosine availability. Enterocytes can both synthesize and take up putrescine [31], however, there was little production of the BA in the absence of the bacterium (Table 2), although a high consumption of agmatine was detected (results not shown) (Table 2), in agreement with the ability of epithelial cells to take up this compound without further metabolism [32]. Moreover, the absence of the human cells had little effect on putrescine synthesis by IOEB 9809 (1330 μM *versus* 1003 μM), in the presence of agmatine and tyrosine. In assays supplemented only with agmatine, a significantly lower level of putrescine was detected in samples containing only bacterial cells (190 μM *versus* 1980 μM). These results indicate that the presence of Caco-2 cells overcomes the need of tyrosine for full induction of the putrescine biosynthetic pathway of IOEB 9809 in DMEM medium. Finally, no synthesis of tyramine by Caco-2 cells was observed in absence of bacteria and a slight but significant increase of the BA levels was observed in the presence of both precursors when either bacteria (220 μM *versus* 320 μM) or co-cultures (180 μM *versus* 230 μM) were analyzed.

**Table 2:** Production of biogenic amines in presence of epithelial cells

**Precursors added**	**Bacteria +Human cells**		**Bacteria**		**Human cells**	
	**Put** (μM)	**Tym** (μM)	**Put** (μM)	**Tym**	**Put** (μM)	**Tym**(μM**)**
**Agm** (4.3 mM)	1980±170^a^	ND	190±80^c^	ND	10±2^d^	ND
**Tyr** (10 mM)	ND	180±9^a^	ND	220±1^ab^	ND	ND
**Tyr** (10 mM) **+ Agm** (4.3 mM)	1330±420^a^	230±9^ab^	1003±41^b^	320±80^b^	7±0^d^	ND

Tyramine (Tym) and putrescine (Put) were detected by RP-HPLC in samples containing DMEM medium supplemented or not with 10 mM tyrosine, 4.38 mM agmatine or both precursors, after 8 h incubation. Cells present during the assay: Bacteria + Human cells: *L. brevis* IOEB 9809 (10^8^ CFU mL^-1^) and Caco-2 cells (10^5^ cells mL^-1^); Bacteria: *L. brevis* IOEB 9809 (10^8^ CFU mL^-1^) and Human cells: Caco-2 cells (10^5^ cells mL^-1^). Results are expressed as the mean ± standard deviation of three independent experiments. ND: not detected. Detection limits: for Put > 2 nM and for Tym > 2.5 nM. Putrescine and tyramine were below the detection limits in the DMEM medium as well as in samples containing either bacteria or Caco-2 cells in absence of the corresponding BA precursor. Differences were assessed by Anova test. Different superscript letters associated with values of the same BA indicate statistically significant differences (P < 0.05).

### Comparison of *L. brevis* IOEB 9809 with *Enterococcus durans* 655

In a previous study [16] we studied the behaviour of *Enterococcus durans* 655 under saliva and gastric stresses as well as in presence of Caco-2 epithelial cells using essentially the same conditions as described in this paper. Our results reveal that the wine *L. brevis* IOEB 9809, like the dairy *E. durans* 655 [16], was able to produce tyramine under saliva and gastric stresses as well as in presence of Caco-2 epithelial cells. In addition, *L. brevis* was able to produce putrescine in all conditions tested. However, unlike *E. durans*[16] an increase of bacterial survival under saliva and mild gastric (pH 5.0-4.0) stresses correlated with transcriptional activation of both BA biosynthetic pathways. Moreover, we found that adhesion levels of *L. brevis* to Caco-2 cells were between 2% and 3%, similar to that detected for *E. durans* 655 (2% or 6% in absence or presence of tyrosine) [16]. We did not detect any influence of the BA biosynthetic pathways on *L. brevis* adhesion capability. However, we have only observed for *L. brevis* an increase of putrescine production in co-cultures of bacteria and epithelial human cells. Thus, it seems that the role of the BA biosynthetic pathways of *Lactobacillus* in the human GIT environment differs from that of *Enterococcus*.

### Potential impact of *L. brevis* IOEB 9809 on human health

With regard to the potential impact of *L. brevis* on human health, our results indicate that during transit through the stomach (1h 40 min in our assay) as well as in contact with Caco-2 cells (8 h) the bacteria could produce around 0.5 mM tyramine (87 mg L^-1^). This should not be harmful for healthy individuals, since an average of 500 mg of orally administrated tyramine is required to increase systolic blood pressure [33]. However, tyramine can be particularly toxic to patients receiving monoamine oxidase (MAO) inhibitors. Gastrointestinal MAO is essential for the breakdown of tyramine and it has been reported that as little as 6 mg of tyramine is sufficient to produce hypertension in humans treated with MAO inhibitors [34]. Ethanol also inhibits MAO. Thus the expected low toxic effect due to low levels of tyramine produced by *L. brevis* during wine fermentation could be potentiated by the simultaneous ingestion of high ethanol content beverages. Moreover, the production of putrescine by this bacterium could be also harmful. The polyamines, including putrescine, play a role in the maturation of the intestine, even when administrated orally [35]. Polyamines administrated orally can act as growth factors with beneficial or detrimental effects, depending on their concentration [36] and there is evidence suggesting that putrescine can cause malignancy in GIT cells [37]. It is estimated that the daily intake of polyamines in the diet is in the range of 350–550 μmol. Thus, the amount of putrescine (around 140 μM) produced by *L. brevis* in 1 h 40 min in the gastric environment seem to be of little concern. However, the 1.3-1.9 mM production of putrescine in the presence of Caco-2 epithelial cells during 8 h, is more worrying, especially if *L. brevis* is able to colonize, even transiently, the small intestine.

## Conclusions

*L. brevis* IOEB 9809 produced both tyramine and putrescine under all conditions in an *in vitro* model that simulated the normal physiological conditions in the human digestive tract, as well as in the presence of Caco-2 epithelial cells. Under mild gastric stress bacterial survival improved in the presence of BA precursors and a synchronous transcriptional activation of the tyramine and putrescine biosynthetic pathways was detected. These results suggest that BA production may be a mechanism that increases bacterial survival under acid stress. The results also indicate that it may be possible for viable cells of *L. brevis* IOEB 9809 to pass from the stomach into the duodenum.

*L. brevis* IOEB 9809 cells were able to adhere to Caco2 cells, which suggests that they may be able to adhere to human intestinal epithelium. However, this would not necessarily guarantee that *L. brevis* IOEB 9809 would colonise the lower intestine as the impact of competition with other resident microorganisms, and the gut's innate defence mechanisms has not been assessed for this organism. High levels of both BA were observed after exposure of the bacterium to Caco-2 cells.

## Methods

### Bacterial strain

*L. brevis* IOEB 9809, isolated from Bordeaux red wine, was obtained from the IOEB strain collection (Institute of Oenology of Bordeaux, ISVV, Villenave d’Ornon, France). The probiotic bacteria *Lactobacillus acidophilus* LA-5 and *Bifidobacterium animalis* subps. *lactis* BB-12 (Chr. Hansen A/S., Hørsholm, Denmark) were also used. All strains were maintained at −80°C in de Man Rogosa Sharpe (MRS) [38] broth (Pronadisa, Madrid, Spain) supplemented with 20% (vol/vol) glycerol.

### Analysis of cell survival under upper digestive tract stress

#### Induction of BA production

Four cultures of *L. brevis* IOEB 9809 were grown at 30°C in MRS initial pH 6.2. One culture was unsupplemented (uninduced), and the other three were supplemented with 10 mM tyrosine (Sigma-Aldrich, St Louis, MO), or 4.38 mM agmatine sulphate (Sigma-Aldrich, St Louis, MO) or both. These concentrations of BA precursors were optimal for production of BA during bacterial growth (results not shown). Pyridoxal phosphate 0.005% (wt/vol) final concentration (Sigma-Aldrich, St Louis, MO) was added to all cultures as coenzyme for decarboxylation reactions. All of the above was performed in triplicate (12 cultures in total). Cells were harvested in the mid-exponential phase (OD_620_ = 0.8, approximately 8 × 10^8^ CFU mL^-1^) by centrifugation, and resuspended in the same volume of the corresponding fresh MRS medium.

#### Digestive tract simulation

To determine the tolerance to saliva and gastric stresses, we modified a previous method [21]. Each of the 12 resuspended cell samples (above) was dispensed in 7 groups of 2.5 ml aliquots. Group 1 (control) was untreated. Group 2 (saliva simulation) 10% (vol/vol) of a sterile electrolyte solution [39] pH 6.5 supplemented with 1% (wt/vol) lysozyme (Sigma-Aldrich, St Louis, MO) was added to each aliquot, and they were incubated for 5 min at 37°C with shaking. Groups 3–7 (gastric environment simulation) 0.3% (wt/vol) pepsin (Sigma-Aldrich, St Louis, MO) was added to saliva simulation followed by acidification with 1 M HCl to pH 5.0, 4.1, 3.0, 2.1 or 1.8 respectively. All aliquots subjected to gastric stress were independently incubated for 20 min, at 37°C with shaking. After the treatments, the bacteria were collected by centrifugation (8.000 × g, 8 min) and cell survival was determined by plate counting on MRS agar. Supernatants were filtered (0.2 μm filters, VWR international, West Chester, PA) and analyzed by reverse-phase high-performance liquid chromatography (RP-HPLC) (see below) for tyramine and putrescine.

### Cell culture and ***in vitro*** adhesion assay

The Caco-2 cell line was obtained from the cell bank of the Centro de Investigaciones Biológicas (Madrid, Spain), and was grown and differentiated as previously described [23]. For the adhesion assay, Dulbecco’s Modified Eagle Medium (DMEM) with L-glutamine (580 mg L^-1^), D-glucose (4500 mg L^-1^) and sodium pyruvate (110 mg L^-1^) pH 8.2 was prepared without L-tyrosine, according to the Invitrogen formulation. DMEM medium was supplemented with the same concentration of L-tyrosine and agmatine sulphate as used for the gastrointestinal experiments. In the adhesion assay experiments, bacteria grown in MRS to the mid-exponential phase (OD_620_ = 0.8) as for BA induction, were centrifuged (10.000 x g, 10 min), washed once with cold phosphate-buffered saline (PBS) pH 7.1 (10 mM Na_2_HPO_4_, 1 mM KH_2_PO_4_, 140 mM NaCl, 3 mM KCl, all purchased from Merck, Darmstadt, Germany) and resuspended in the same DMEM medium supplemented, or not, with tyrosine, agmatine or both. Bacterial suspensions were added to Caco-2 intestinal cells in a final volume of 0.1 mL and a final concentration of 1.25 x 10^7^ CFU mL^-1^ (ratio 1:100, Caco-2 cells to bacteria) and incubated at 37°C for 1 h. Unbound bacteria were then removed by washing three times with 0.2 mL of PBS at pH 7.1. Some wells, unwashed, were used as control. Cell cultures were then resuspended in 0.1 mL of PBS and detached by adding 0.1 ml of 0.05% trypsin-EDTA (Gibco, Carlsbad, CA). After incubation at 37°C for 10 min, the detachment reaction was interrupted by adding 0.1 mL of cold PBS. The number of total and adhered bacteria was determined by serial dilution and quantitation on agar plates as for viable counts. The adhesion percentage was calculated by comparing the number of CFU from three washed wells with those from control wells. Every experiment was performed in triplicate.

### RP-HPLC determination of BA

Pre-column dabsyl chloride manual derivatisation was performed for BA detection. The derivatisation reaction was carried out as described by Krause et al. [40]. 10 μl of the dabsylated supernatants were used for injection. HPLC analysis was performed using an Alliance 2795 system (Waters, Milford, MA) equipped with a Waters Nova-Pack C_18_ column (150 × 3.9 mm 4 μm particle size). Dabsylated amino acids and amines were eluted using the gradient described by Krause et al. [40]. Detection was carried out by a Waters 2996 Photodiode array detector at 436 nm.

### RNA extraction and Real Time PCR analysis

Transcriptional analysis was performed after 20 min gastric stress simulation. Control and samples mimicking gastric stress at pH 5.0, were analyzed in the presence or absence of biogenic amine precursors. Total RNAs were extracted from 2 × 10^9^ cells using the FastRNA pro blue kit (Qbiogene, Montreal**,** QC) following the manufacturer’s instructions. Cells were lysed mechanically with a Hybaid Ribolyser for 30 s. The RNAs' quantity and quality was determined by spectrophotometry, and their integrity was assessed by visualization of the rRNA bands on 1.2% agarose gels. Absence of chromosomal DNA was confirmed by quantitative real-time PCR.

cDNAs were synthesized using 0.8 μg of total RNA and Quantitect Reverse Transcription (Qiagen, Hilden**,** Germany) which included a DNase treatment and reverse transcription.

Primers for real time PCR were designed to have a length around 20 bases, a GC content of approximately 50% and a Tm around 60°C. OligoPerfect Designer software (Invitrogen, Carlsbad, CA) was used to select primers sequences. Secondary structures and dimer formation were predicted using Oligo Analyzer 3.0 software (Integrated DNA Technologies, Coralville**,** IA). Primers were purchased from Sigma-Aldrich (St Louis, MO).

Real time PCR was performed using an Applied Biosystems 7300 Real-Time PCR System. The *tuf* gene of *L. brevis,* encoding elongation factor Tu, was used as internal control for the analysis of *tyrDC* and *aguA1* genes expression, as previously described for *Streptococcus thermophilus*[41]. Standard curves for both the internal-control and target genes were obtained by amplifying serial dilutions (ratio, 1:10) of the target sequences. Additionally, data were normalized in function of the amount of total RNA, according to Torriani et al. [42].

The amplifications were carried out in 20 μl reactions, by adding 5 μl of 1:20 diluted cDNA, to a real-time PCR mix containing Power SYBR Green PCR Master Mix (Applied Biosystems, Foster City, CA), according to the manufacturer’s instructions, and 100 nM of each primer. The *tyrDC* (EMBL accession number LVIS_2213) specific cDNA was amplified with the TDC_F (5^′^-TGAGAAGGGTGCCGATATTC-3^′^) forward and the TDC_R (5^′^-GCACCTTCCAACTTCCCATA-3^′^) reverse primers. The *aguA1* (EMBL accession number LVIS_2208) specific cDNA was amplified with the AGUA1_F (5^′^-TCTTGAAAATGCGACAGACG-3^′^) forward and the AGUA1_R (5^′^-TCCAACGTAGCCTGAGCTTT-3^′^) reverse primers. The TUF_F (5^′^-AGGCGACGAAGAACAAGAAA-3^′^) forward and the TUF_R (5^′^-CGATACGACCAGAAGCAACA-3^′^) reverse primers were used to amplify the *tuf* (EMBL accession number LVIS_1389) specific cDNA.

Thermal cycling was as follows: initial denaturing at 95°C for 5 min followed by 35 cycles at 95°C for 15 s and 60°C for 35 s. The amplicons' lengths were 141 bp, 240 bp and 159 bp for the *tyrDC*, *aguA1* and *tuf* genes respectively and their specificity was checked by melting curve analysis.

A threshold cycle value (CT) was determined with a base line settled automatically. The relative expression level of genes was calculated by the 2^-∆∆ct^ method, using unstressed, and unsupplemented with BA precursors, total RNA as calibrator. The relative expression of *tyrDC* and *aguA1* during the other experimental conditions was quantified as n-fold differences with respect to the calibrator.

Real-time PCRs were performed in duplicate for each sample of cDNA, including a negative control in each run. Data were expressed as the mean of three independent experiments.

### Confocal laser scanning microscope

Samples from each gastric stress condition were analyzed by confocal laser scanning microscopy (model TCS-SP2-AOBS, Leica Microsystems GmbH, Wetzlar, Germany), after staining with SYTO9 and propidium iodide (LIVE/DEAD® *Bac*Light™ bacterial viability kit, Molecular Probes, Inc. AA Leiden, The Netherlands) to differentiate the cells as a function of compromised membranes. Confocal illumination was provided with a X63 magnification objective and numerical aperture of 1.40–0.60 and by Argon laser (488 nm laser excitation) with a long pass 520–565 nm filter (for green emission) and long pass 630–685 nm filter (for red emission). Image analysis was performed using FRET and FRAP software (Leica Microsystems GmbH, Wetzlar, Germany).

### Statistical analysis

Anova statistical tests were used to evaluate the consistency of the data.

## Abbreviations

Agm: Agmatine; BA: Biogenic amines; GIT: Gastro intestinal tract; LAB: Lactic acid bacteria; RP-HPLC: Reverse-phase high-performance liquid chromatography; Tym: Tyramine; Tyr: Tyrosine; Put: Putrescine.

## Authors’ contributions

**PR** carried out the transcriptional analysis, help to perform the *in vitro* GI tract system and drafted the manuscript. **AR** and **MF** carried out the biogenic amines detection and quantification and performed the statistical analysis. **PFP** set up the *in vitro* GI tract, confocal microscope analysis and the adhesion assay experiments. **GS**, **PL** and **PaLu** participated in the design of the study, coordination and helped to draft the manuscript. All authors read and approved the final manuscript.
